# Nematicidal Activity of the Endophyte *Serratia ureilytica* against *Nacobbus aberrans* in Chili Plants (*Capsicum annuum* L.) and Identification of Genes Related to Biological Control

**DOI:** 10.3390/plants10122655

**Published:** 2021-12-03

**Authors:** Arnoldo Wong-Villarreal, Erick Williams Méndez-Santiago, Olga Gómez-Rodríguez, Liliana Aguilar-Marcelino, Daniel Cerqueda García, José Q. García-Maldonado, Victor M. Hernández-Velázquez, Gustavo Yañez-Ocampo, Saúl Espinosa-Zaragoza, Sandra I. Ramírez-González, Diana Sanzón-Gómez

**Affiliations:** 1 División Agroalimentaria, Universidad Tecnológica de la Selva, Ocosingo 29950, Mexico; 2 Centro de Investigación en Biotecnología, Universidad Autónoma del Estado de Morelos, Cuernavaca 62209, Mexico; Erick_williams.1994@hotmail.com (E.W.M.-S.); vmanuelh@uaem.mx (V.M.H.-V.); 3 Programa de Fitopatología, Colegio de Postgraduados-Campus Montecillo, Texcoco 56230, Mexico; 4 Centro Nacional de Investigación Disciplinaria en Salud Animal e Inocuidad, INIFAP, Jiutepec 62550, Mexico; aguilar.liliana@inifap.gob.mx; 5 Red de Manejo Biorracional de Plagas y Vectores, Instituto de Ecología, A.C.—INECOL, Xalapa 91073, Mexico; daniel.cerqueda@inecol.mx; 6 Centro de Investigación y de Estudios Avanzados del Instituto Politécnico Nacional, Unidad Mérida, Mérida 97310, Mexico; jose.garcia@cinvestav.mx; 7 Laboratorio de Edafología y Ambiente, Universidad Autónoma del Estado de México, Toluca 50000, Mexico; yanezg0206@gmail.com; 8 Facultad de Ciencias Agrícolas, Universidad Autónoma de Chiapas, Huehuetán 30660, Mexico; saulez1@gmail.com; 9 Laboratorio de Agrotecnologías, Centro Universidad Empresa, Universidad Autónoma de Chiapas, Tuxtla Gutiérrez 29050, Mexico; sanir@yahoo.com; 10 División Ciencias de la Vida, Departamento de Agronomía, Campus Irapuato-Salamanca, Universidad de Guanajuato, Irapuato 36500, Mexico; dianas7@yahoo.com.mx

**Keywords:** biocontrol, rhizobacteria, false root knot, vegetables

## Abstract

The genus *Serratia* is widely distributed in soil, water, plants, animals, invertebrates, and humans. Some species of this genus have antifungal, antibacterial, and nematicidal activity. In this work, the nematicidal activity of the endophytic strain of *Serratia* sp. in chili, *Capsicum annuum* L., is reported, where at a bacterial concentration of 4 × 10^9^ cel/mL, the penetration of nematodes into the roots significantly decreased by 91 and 55% at 7 and 21 days after inoculation. This bacterial concentration also significantly decreased the number of galls, eggs, egg masses and reproduction factor produced by *Nacobbus aberrans* in Chili plants, with respect to the control where this bacterial strain was not applied. In the analysis of the genome of the strain, based on average nucleotide identity (ANI), the isolate could be affiliated to the species *Serratia ureilytica*. The size of the genome is 5.4 Mb, with a 59.3% content of GC. Genes related to the synthesis of chitinases, siderophores, proteases C, serralisins, hemolysin, and serrawettin W2 that have been reported for biocontrol of nematodes were identified in the genome. It is the first report of *Serratia ureilytica* with nematicidal activity. Based on these results of nematicidal activity, this strain can be evaluated in the field as an alternative in the biocontrol of *Nacobbus aberrans* in chili cultivation.

## 1. Introduction

The false root-knot nematode *Nacobbus aberrans* is a species located within the top 10 phytoparasitic nematodes worldwide [1], and within the most economically important groups in Mexico in the crops of chili, tomato, and bean [2]. When feeding, the sedentary females of *N. aberrans* induce the formation of galls in the roots, which block the vascular system resulting in a nutritional imbalance and poor water absorption, impacting on crop production [3,4]. In Mexico, losses caused by *N. aberrans* are reported in tomato yields of 55%, chili 50–70%, and beans 36% [2,3,4,5]. In general, chemical control of phytonematodes has been the main management strategy, but this generates contamination problems due to its persistence in the environment, causing animal and human health problems. Additionally, damage to beneficial organisms has been observed [6,7]. Therefore, in recent decades nematode management has been focused on the use of different control methods, such as biocontrol that uses different enemies or natural antagonists such as fungi and bacteria [6].

Numerous species of soil bacteria located in the rhizosphere stimulate plant growth and reduce the nematode population through antagonistic behavior; collectively they are known as plant growth-promoting rhizobacteria (PGPR), among which are the genera *Bacillus*, *Pseudomonas*, and *Serratia* [8,9]. Bacteria-nematode interaction can reduce nematode populations by the effect of enzymes, toxins, and secondary metabolites as products of antagonist rhizobacteria. Hydrolytic enzymes, mainly proteases, collagenases, and chitinases, have been proposed to have nematicidal activity due to the degradation of nematode components in their different stages of development [9]. Among the first works in which the use of the *Serratia* bacteria is reported is that of Mercer et al. [10], who used chitinases produced by *Serratia* spp. with a deleterious effect on *Meloidogyne hapla* eggs. Currently, nematicidal activity of *Serratia* spp. has been reported both under in vitro and in vivo conditions against some plant-parasitic nematodes. Zhao et al. [11] used bacteria of the *Pseudomonas*, *Bacillus*, and *Serratia* genera against *Meloidogyne incognita* eggs under in vitro conditions and reported the highest larvicidal and ovicidal effect of *Serratia proteamaculans* with 99.1% mortality of second stage juveniles (J2) and 61.11% egg mortality. Paiva et al. [12] report nematicidal activity of *Serratia* sp. against *Bursaphelenchus* spp. by attributing the presence of proteases in the supernatant. On the other hand, Almaghrabi et al. [8] evaluated the effect of six PGPRs: *Pseudomonas putida*, *P. fluorescens*, *S. marcescens*, *B. amyloliquefaciens*, *B. subtilis*, and *B. cereus* on the growth of tomato plants as well as against the root-knot nematode *M. incognita*, where the highest dry weight, height, and number of fruits per plant were obtained in plants treated with the bacterium *S. marcescens*, which corresponded to the lowest number of J2 in the soil, galls, and eggs masses per root. Similarly, the prodigiosin pigment extracted from *S. marcescens* has been used with a nematicidal effect on the hatching of the J2 from the egg of the PPN *Radopholus similis* and *M. javanica* [13]. Abebe-Akele et al. [14] reported the genomes of *Serratia marcescens* DB11, *S. proteamaculans* 568, and *Serratia* sp., isolated from the nematode *Caenorhabditis briggsae*, where they identified genes that are related to entomopathogenic activity against *Galleria mellonella* larvae. The genome of *Serratia* sp. YD25, isolated from the rhizosphere of tobacco cultivation, bore genes related to the production of serrawettin W2 and prodigiosin metabolites important for biocontrol [15].

According to these antecedents, the nematicidal activity of *Serratia* sp., isolated from nodules of *Mimosa pudica* from the state of Chiapas, Mexico, was evaluated in vitro. A mortality of 88.8% was recorded in juveniles of the second stage of *N. aberrans*, and 100% in infective larvae (L3) of the sheep parasitic nematode *Haemonchus contortus* and free-living *Panagrellus redivivus* [16]. Therefore, the present research aims to report the genome of a strain of *Serratia* with nematicidal activity against *N. aberrans* in chili plants, for the identification of genes associated with this activity and contribute to the sustainable management of this phytoparasitic nematode given its economic impact on protected agriculture.

## 2. Results

### 2.1. Nematode Penetration

**Figure 1:**
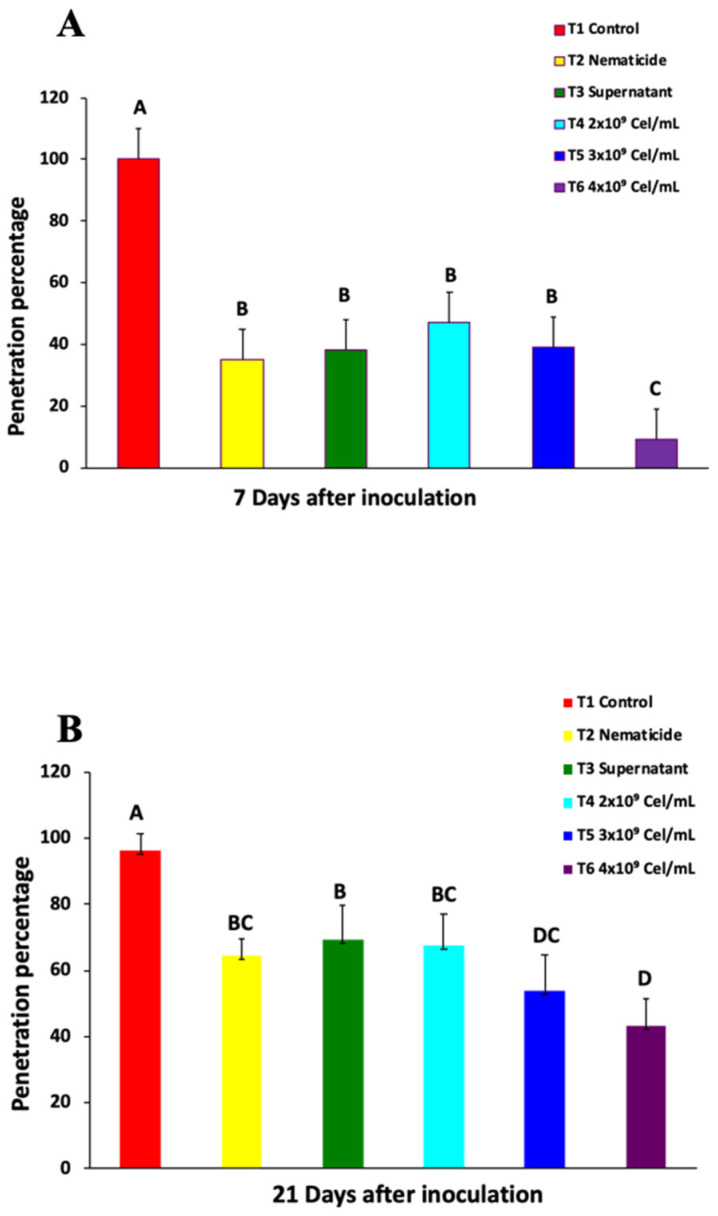
Penetration percentage of nematodes per root in chili plants inoculated with *Nacobbus aberrans* (**A**) At 7 days after inoculation. Coefficient Variation = 8.92%. (**B**) At 21 days after inoculation. Coefficient Variation = 6.96%. Treatments with the same letter do not have significant differences.

The percentage of nematodes that penetrated the root of each plant at 7 and 21 days after inoculation (dai) with *N. aberrans* is presented in Figure 1A,B. The plants with the highest percentage of nematode penetration on both evaluation dates were recorded in the treatment where only the nematode (Na) was inoculated, and with significant differences (*p* < 0.05) with respect to the different treatments evaluated. At 7 days, no significant differences were recorded between the treatments of the plants treated with nematicide, the supernatant, and *Serratia* sp. with the two lowest concentrations (Se-2 and Se-3 treatments with 2 × 10^9^ and 3 × 10^9^ cel/mL, respectively). The aforementioned treatments (iu, supernatant, Se-2, and Se-3) presented a significant reduction (*p* < 0.05) with respect to the Na treatment between 53 to 65%, and for the highest concentration (Se-4, 4 × 10^9^ cel/mL) with a value of 91%. This last treatment (Se-4) had the lowest penetration percentage and significantly different from the evaluated treatments. At 21 dai there was a similar behavior to 7 dai, except for the Se-3 treatment, which presented a significantly (*p* < 0.05) lower percentage of nematodes that penetrated with respect to the plants treated with the supernatant, and without differences with respect to at the highest concentration of the bacterium Se-4 (4 × 10^9^ cel/mL). The treatments in which the plants were treated with nematicide, supernatant, and the lowest concentration of the bacteria registered a significant reduction (*p* < 0.05) compared to the control (Na) from 28 to 45%, and for the treatments with *Serratia* (Se-3, and Se-4) with reductions of 45 and 55%. In general, the lowest percentage of nematodes was registered in the Se-4 treatment with a reduction of 91% at 7 dai and 55% at 21 dai with respect to the Control (Na).

### 2.2. Galls, Egg Masses, Eggs, and Reproduction Factor

**Figure 2:**
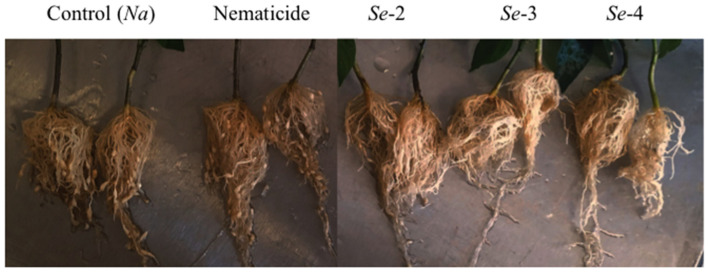
Galling in chili plants cv. Tampiqueño 42 days after inoculation with 1000 J2 of *N. aberrans* in the growth chamber. Na, plants inoculated only with the nematode; Nematicide, plants with Nemacur^®^; plants with the bacteria at different concentrations: Se-2 (2 × 10^9^ cel/mL), Se-3 (3 × 10^9^ cel/mL), and Se-4 (4 × 10^9^ cel/mL).

The number of galls, egg masses, eggs, and reproduction factor are presented in Table 1. The control (Na) was significantly (*p* < 0.05) different, presenting the highest number of galls with respect to the plants treated with the nematicide and the concentration of 4 × 10^9^ cel/mL of *Serratia* sp. (Se-4), as is evident in Figure 2, greater number of egg masses and reproduction factor with respect to the lower and higher concentration of the bacteria 2 × 10^9^ cel/mL, Se-2 and 4 × 10^9^ cel/mL Se-4, and the highest number of eggs with respect to all the treatments evaluated (Table 1). Treatment Se-4 registered the lowest number of galls, masses of eggs, and eggs with reductions of 47%, 62%, and 91%, respectively, with respect to the control (Na) when considering this treatment (Na) with a value of 100%, and without significant differences with respect to the application of the nematicide. Furthermore, the Se-4 treatment had the lowest reproduction factor, equivalent to 1.26, while in the control it was 3.3 (Table 1).

### 2.3. Genome Assembly and Annotation

According to the average nucleotide identity (ANI) analysis of the genome of strain UTS and closely related genomes from GenBank, the highest ANI value was 98.96% with *Serratia ureilytica* Niva51(GCA_013375155.1). This value is higher than the widely accepted threshold range (95–96%) for species demarcation [17,18]. The *Serratia ureilytica* UTS strain contains 134 contigs with a size of 5.40 Mbp (Table 2); the genome includes 5303 coding sequences (CDS) and 93 RNA genes. The mean GC content is 59.3%, similar to *S. marcescens* strains; 954 hypothetical proteins and 4394 proteins with assigned functions were also reported (Table 2).

**Figure 3:**
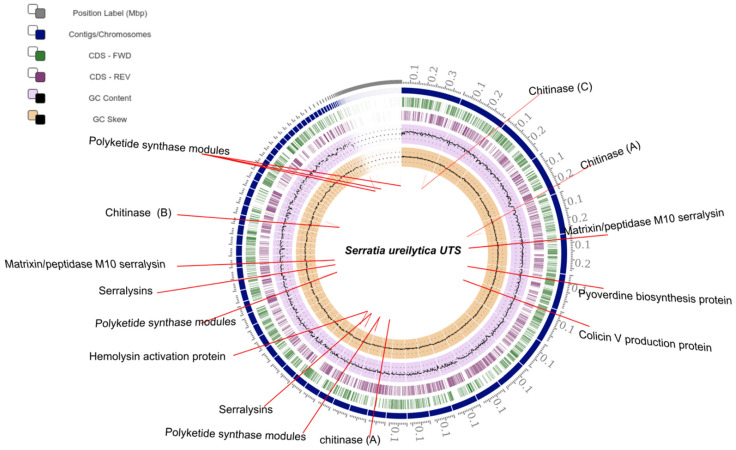
Circular representation of the genome of Serratia ureilytica UTS. From outside to inside: the first circle shows the contigs of the genome; the second and third circles show genes in the forward and reverse strands, respectively; the fourth and fifth circles, GC content and GC skew.

**Figure 4:**
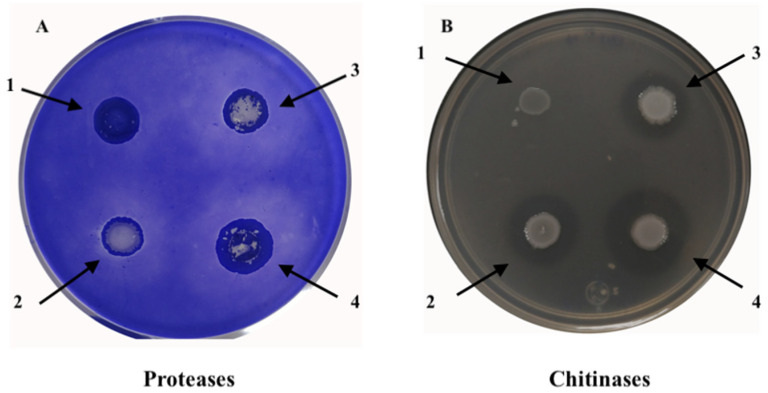
(**A**) Secretion of extracellular protease and (**B**) chitinase activity from *Serratia ureilytica* UTS. The culture medium used for the detection of extracellular protease contains skim milk and for chitinase it contains 1% colloidal chitin, respectively. The protease or chitinase activity is observed by the formation of halo around the bacterial growth after incubation for 16–24 h at 30 °C. The inoculated spots in the culture medium correspond to different optical densities quantified at 600 nm of the culture of *Serratia ureilytica* UTS 2: (0.4), 3 (0.6) and 4 (0.8), the inoculated point that does not present halo, corresponds to the control negative, (1) *Escherichia coli*.

Genome sequence analysis revealed the presence of homologous proteins involved in the biosynthesis of bacteriocin pyoverdin, colicin V, hydrogen cyanide, hemolysin, serrawettin W2, proteases, chitinases A, chitinases B and chitinases C (Figure 3, Table 3). The results of the enzyme activity assay also indicated that *S. ureilytica* UTS possesses the ability to secrete extracellular protease and chitinase (Figure 4).

### 2.4. Protease and Chitinase Degradation Assays

Chitinase activity was detected by observing the formation of halos around *S. ureilytica* UTS in the medium with colloidal chitin; Protease activity was observed by the formation of halos in a medium containing skim milk. In Figure 4 different sizes of halos can be observed both in chitinase and protease activity, this is due to the fact that different optical densities of the culture were inoculated: 1 (0.4), 2 (0.6) and 3 (0.8); in the inoculum where no halo is observed is the negative control: 4 (Escherichia coli)

### 2.5. Figures and Tables

(T1) Plants only with the nematode (Na), (T2) Plants treated with commercial nematicide (Nematicide), (T3) Plants treated with the bacterial growth supernatant (Supernatant) (T4), Plants treated with *Serratia* sp. (Se-2, 2 × 10^9^ cel/mL), (T5) Plants treated with *Serratia* sp. (Se-3, 3 × 10^9^ cel/mL), and (T6) Plants treated with *Serratia* sp. (Se-4, 4 × 10^9^ cel/mL). The commercial nematicide used was Nemacur^®^ 38.80% CE (Fenamiphos) applied at a dose of 2%. The values (mean ± SD) in each column followed by the same letter are not significantly different.

## 3. Discussion

The application of the bacteria *Serratia* sp. to the inoculation with the juveniles of the second stage (J2) of *N. aberrans* in chili seedlings showed a nematicidal effect at the highest concentration evaluated (Se-4, 4 × 10^9^ cel/mL) (Figure 1). It presented significant differences (*p* < 0.05) both in the percentage of nematodes that penetrated the root and in the number of galls, egg masses, eggs, and reproduction factor with respect to the plants that were inoculated only with the nematode (Na, Control) (Table 1, Figure 1). In the assessment of the nematicidal capacity of this strain of bacteria under in vitro conditions, an 88.8% mortality of J2 of *N. aberrans* was recorded [16], an effect confirmed in vivo under the conditions evaluated in the present investigation. The species *Serratia* is reported as an efficient biological chitin degrading bacterium [19]. Within the characterization of the strain used, chitinase production was recorded (specific activity of 13,999 U/mg) [16]. The main component of the nematode cuticle is collagen, without the presence of chitin [20,21,22]; however, there are reports that indicate a nematicidal effect of chitinases both in solution and those produced by different species of bacteria, among which is *Serratia* sp. when using a chitinase solution. Millew and Sands [23] observed structural changes in the cuticle of juveniles (J2) of *Tylenchorhynchus dubius* incubated after 2.5 h, and a reduction in the movement of individuals above 75%. Likewise, Sánchez [24] used a chitosan solution at concentrations of 2000 and 1500 ppm and recorded a mean immobilization of 145.9 and 140.5 in juveniles of the second stage (J2) of *N. aberrans* at 72 h, respectively, compared to a mean of 51.9 when using water. The use of a chitinase solution can be comparable to when the supernatant in which the bacteria grew was used, a treatment in which a significantly (*p* < 0.05) lower percentage of nematodes that penetrated the chili root was recorded at 7 and 21 dai (9.9 and 6.9%, respectively), and in the number of eggs per gram of root (168) at the end of the evaluation, compared to the control. In relation to the penetration of nematodes at 21 dai, Sánchez [24] also reports a lower percentage of individuals in CM-334 chili roots treated with a chitosan solution at 2000 ppm and inoculated at the same time with *N. aberrans* J2.

The greatest nematicidal effect was evidenced when the highest concentration (4 × 10^9^ cel/mL) of *Serratia* was used; This effect is perhaps not only related to the chitinolytic activity of the strain [16], but also to the activity of other enzymes such as proteases. In general, rhizobacteria are attributed a nematicidal effect due to the activity of enzymes such as proteases, collagenases, and chitinases, which can act on different chemical components of nematodes in different stages [9]. Hegazy et al. [25] report a high percentage of juvenile mortality (J2) of *M. incognita* (96%) after 24 h with *S. marcescens* subsp. marcescens due to a high activity of chitinases and proteases, and they inferred that the activity of chitinases was more relevant than proteases in the mortality of nematodes. However, Paiva et al. [12] attributed the death of the nematode *B. xylophilus* to the production of proteases of *Serratia* sp., a similar effect was observed in the treatments where the bacteria were used at different concentrations (treatments Se-2, Se-3, and Se-4). The lowest number of nematodes that penetrated the root of the chili plants was recorded, with the treatment with the highest concentration of cel/mL of *Serratia* sp., (Se-4), significant differences were observed (*p* < 0.05) with respect to the treatment where the chemical nematicide is applied (Table 1).

Meanwhile, the use of the nematicide registered a significantly (*p* < 0.05) lower number of galls and eggs with respect to the control (Table 1). In contrast, the use of *Serratia* sp., (Se-4, 4 × 10^9^ cel/mL) registered the lowest values of number of galls, egg masses, eggs, and reproduction factor, this last variable with a value of 1.26 and with significant differences (*p* < 0.05) with respect to the control with 3.31 (Table 1). This represents that there will be a higher level of inoculum in the rhizosphere of this last treatment. Our results are added to those reported by other studies in which an enzymatic activity of the species *Serratia* sp. with nematicidal effect against some species of plant-parasitic nematodes are reported. Almaghrabi et al. [8] treated tomato plants with *S. marcescens* and inoculated with *M. incognita* and recorded a lower number of galls and egg masses. In addition, in tomato, Zhao et al. [11] treated the seeds with *S. marcescens* and observed a lower number of galls and juveniles of *M. incognita* in the soil. For their part, Zeinat et al. [26] recorded a lower number of galls in *Vicia fava* plants inoculated with *Meloidogyne* sp. and treated with *S. marcescens*. Furthermore, the use of *Serratia* sp. against *N. aberrans* can be favored by the eating habits of this species of nematode, since it behaves as a migratory endoparasite in the stages of J2, J3, J4 and vermiform immature female when re-entering the root of its hosts, and only establishes itself as a sedentary endoparasite in the female stage, being exposed to the microorganisms of the rhizosphere at various stages within its life cycle [27,28]. Meanwhile *Meloidogyne* sp., which is also a root-knot nematode, penetrates the J2 to the root and establishes itself in it [28]. With the same population of *N. aberrans* that was used in the present study, Cristóbal et al. [29] point out that juveniles J3 and J4 survive in the soil under field conditions and without hosts for 12 months, conserving their infectivity when inoculated in tomato plant roots. This condition makes them vulnerable to the enzymatic activity of different species of bacteria and fungi. The nematicidal activity of this strain in chili plants generated interest in identifying genes that may be involved in the biosynthesis of metabolites involved in biocontrol.

Genome analysis allowed the strain to be identified as *S. ureilytica* with 98.93% identity, based on average nucleotide identity (ANI), this strain does not have a plasmid; However, strains such as *S. ureilytica* CC119 (PRJNA487218) and *S. ureilytica* JBIWA004 (PRJNA725976) have been reported that possess plasmid. Some strains of *Serratia* produce metabolite prodigiosin related to nematicidal activity against juvenile states of *Radopholus similis* and *M. javanica*, as well as egg hatching [15,16,17,18,19,20,21,22,23,24,25,26,27,28]. However, in this strain this pigment is not present, so the nematicidal activity must be related to the production of chitininase enzymes as reported by Hegazy et al. [25], where the activity of this enzyme produced by *S. marcescens* subsp. marcescens has 96% mortality in juveniles (J2) of the nematode *M. incognita.*

Another molecule that is associated with nematicidal activity is Serrawettin W2, a biosurfactant of lipopetidic nature [30]. The genes that encode for this molecule have been identified in *S. ureilytica* UTS (Table 3), this agrees with that reported by Marques-Pereira et al. [31], where they identified these genes in different *Serratia* species. Genes associated with the production of protease enzymes in the genome were also identified. These enzymes have been detected in *Serratia* sp. A88copa13, where they were related to nematicidal activity against *Bursaphelenchus tusciae*, *B. mucronatus*, and *B. conicaudatus* nematodes (Paiva et al. [12]. Although the purification of proteases and chitinases in the strain of *S. ureilytica* TKU013 have been reported for the degradation of squid pen; as well as the production of Serratiw w2 in other strains of *S. ureilytica* [32]. No reports of *S. ureilytica* with nematicidal activity were found, so it is considered that the *S. ureilytica* UTS strain is the first report of this species with nematicidal activity.

## 4. Materials and Methods

### 4.1. Plant Material

Chili type serrano cv. Tampiqueño, susceptible to *N. aberrans*, was used. The chili seeds were disinfected with 0.5% sodium hypochlorite for 1 min and rinsed with sterile distilled water. For their germination, they were placed on sterile paper towels moistened with sterile distilled water in germinating boxes at 28 °C. The seedlings were transplanted into pots with 25 cc of sterile fine sand (one seedling per pot), and kept in a growth chamber at a temperature of 27 ± 1 °C, with a photoperiod of 14 h with a light intensity of 6768 lux (Fluorescent light). It was fertilized weekly with a nutrient solution (3.15 g of Nitrofoska^®^ 12-12-12-2 per liter of sterile water).

### 4.2. Obtaining and Inoculation of Serratia sp. (Se)

The preparation of the inoculum of the *Serratia* strain was carried out in PY liquid culture medium containing the following ingredients (g/L): tryptone casein (5), yeast extract (3), calcium chloride (0.1) and with the following growth conditions: 30 °C, 200× rpm for 72 h. The concentrations of each inoculum were adjusted to an optical density (OD) of 0.5 (2 × 10^9^ cel/mL), 0.75 (3 × 10^9^ cel/mL), and 1 (4 × 10^9^ cel/mL) at 600 nm. The chili plants were inoculated with each concentration of the bacteria 21 days after transplantation and a second inoculation four days after the first.

### 4.3. Obtaining Inoculum and Inoculation with N. aberrans (Na)

The inoculum of *N. aberrans* was obtained from galled roots of tomato grown in naturally infested soil at Colegio de Postgraduados, Montecillo campus, State of Mexico, Mexico, and kept in greenhouse tomato plants (cv. Rio Grande). Nematode eggs extraction was carried out following the methodology described by Vrain [33], and they were incubated at 28 ± 1 °C in Petri dishes with sterile distilled water, until hatching of the second stage juveniles (J2). Each chili plant with two to three pairs of true leaves was inoculated with 1000 J2 of *N. aberrans* five days after inoculation with *Serratia* sp.

### 4.4. Experiment Establishment and Evaluation

The experiment was established during 2018 and 2019. The experimental design was completely randomized and consisted of six treatments with 22 pots/treatment. Treatments: (1) Na (Control, plants only with the nematode), (2) Nematicide (plants treated with nematicide), (3) Supernatant [plants treated with the supernatant of the liquid culture medium PY (g/L: casein tryptone, 5; yeast extract, 3; and calcium chloride, 0.1), where the bacteria grew], (4) Se-2 (plants treated with Se at an optical density (OD) of 0.5 with a total of 2 × 10^9^ cel/mL), (5) Se-3 (plants treated with Se at an OD of 0.75 with 3 × 10^9^ cel/mL), and 6) Se-4 (plants treated with Se at an OD of 1.0 with 4 × 10^9^ cel/mL). The commercial nematicide used was Nemacur^®^ 38.80% CE (Fenamiphos) applied at a dose of 2%.

The nematicidal activity of *Serratia* sp. On *N. aberrans* was evaluated 7, 21, and 42 days after inoculation (dai) with the nematode. At 7 and 21 dai, the number of individuals per root of each plant was counted under light microscopy in six plants per treatment, and the results were expressed as a percentage. Plants roots were stained using the sodium hypochlorite-acid fuchsin method [34]. The number of individuals per root was counted using a stereoscopic microscope (4×, 10×).

At 42 dai of the nematode, the number of galls, egg masses, and eggs/g of root in 10 plants per treatment were counted. In addition, with the number of eggs of each root, the reproduction factor (RF) was calculated where RF = (Final population/initial population) (100). Egg extraction was carried out according to Vrain [33]. To quantify the egg masses, the roots were immersed in a solution of Phloxin B (0.15 g/L of water) for 20 min so that the egg masses were stained red and counting was facilitated.

### 4.5. Statistical Analysis

The data on the percentage of penetration of nematodes, number of galls, egg masses, eggs/g of root, and reproduction factor were subjected to an analysis of variance (ANOVA) using the general linear model, and a comparison of means with the Tukey’s test and the hovtest to see the homogeneity in the treatments with a significance level of *p* ≤ 0.05, with the use of the “Statistical Analysis System” program (SAS version 9.0).

### 4.6. DNA Extraction, Library Preparation, and Sequencing

Genomic DNA was extracted from freshly cultivated cells of the strain using the ZR Fungal/Bacterial DNA Kit kitTM, according to the manufacturer’s instructions. DNA was quantified with a Qubit” 3.0 Fluorometer using the Qubit dsDNA HS Assay Kit (Life Technologies, Carlsbad, CA, USA). The library was created from 1 ng of DNA using the Nextera XT DNA Library Prep Kit (Illumina, San Diego, CA, USA), and purified using AMPure XP magnetic beads, according to the supplied protocol (Beckman Coulter Genomics, Brea, CA, USA). The correct size of the library was verified on an Advanced QIAxcel (QIAGEN, Germantown, TN, USA). Paired-end sequencing (2 × 150 bp) was performed at CINVESTAV Mérida with the MiSeq platform (Illumina, San Diego, CA, USA) using a 300-cycle MiSeq Reagent Micro Kit v2. Raw sequence data produced in this study was deposited in NCBI under the Bioproject accession number PRJNA759051.

### 4.7. Genome Assembly and Annotation and Taxonomic Classification

The paired-end (2 × 150) raw Illumina reads were trimmed for adapters and filtered at a Q score > 30. De novo assembly was performed with SPAdes (v. 3.12) [35]. Assembly was exported to the PATRIC platform [36], and annotation was performed with RAST (Rapid Annotations using Subsystems Technology) [37]. Taxonomic identification was performed with the GTDB-Tk software, which uses a set of 120 marker genes to place the draft genome within the curated Genome Taxonomy Database references tree and FastANI (Average Nucleotide Identity) [38].

### 4.8. Homologous Identification

A reciprocal blast between amino acid sequences of the *Serratia ureilytica* UTS draft genome and the amino acid sequences of *Serratia marcescens* complete genome (MJVB00000000, MJVC00000000) was performed using blastp with a threshold of 90% of similarity and e-value < 0.00001.

### 4.9. Accession of the Genome Sequences

The data from this Whole Genome Shotgun project was deposited in GenBank with Accession JAIQCT000000000, Bioproject PRJNA759051 and Biosample access number SAMN21162916

### 4.10. Protease and Chitinase Degradation Assays

The bacteria *Serratia* strain was seeded in triplicate in solid NBRIP (glucose, 1%; Ca_3_(PO_4_)_2_, 0.5%; (NH_4_)_2_SO_4_, 0.01%; MgSO_4_·7H_2_O, 0.025%; KCl, 0.02%; MgCl_2_·6H_2_O, 0.5%; Congo red, 2.5 mg/mL; agar, 1.8%) culture medium supplemented with 1% (*w/v*) of colloidal chitin (Sigma-Aldrich Corp, Darmstadt, Alemania.) as sole carbon sources [16]. The bacteria isolates were checked for their production of chitinase by observation of clear zone around the colonies after four days incubation at 30 °C. To determine the protease activity, the *Serratia* strain was seeded in triplicate in the nutritive culture medium supplemented with 1% (*w*/*v*) skimmed milk; the protease activity was observed by the appearance of a clear halo around the bacterial growth [39].

## 5. Conclusions

The *S. ureilytica* UTS strain used in the present research showed a nematicidal effect against *N. aberrans* in chili plants, type serrano cv. Tampiqueño that were inoculated. The inoculated plants significantly decreased the number of nematodes (J2) in the root, number of galls, egg masses, eggs, and reproduction factor. This nematicidal activity may be related to the genes identified in the genome of the strain that have been reported with biocontrol activity. The use of this bacterial strain has potential in the sustainable management of the false root knot *N. aberrans*.

## Figures and Tables

**Table 1:** Number of galls, egg masses, eggs/g of root, and reproduction factor (RF) in chili plants cv. Tampiqueño 42 days after inoculation with 1000 J2 of *N. aberrans* in the growth chamber.

Treatments	Galls	Egg Masses	Eggs	RF (%)
T1	41.0 ± 1.4 a	16 ± 1.6 a	584 ± 60 a	3.31 ± 0.45 a
T2	22.5 ± 1.1 b	9 ± 1.3 ab	145 ± 12.6 cd	2.28 ± 0.64 ab
T3	29.0 ± 0.9 ab	9 ± 1.1 ab	168 ± 18.8 b	2.29 ± 0.56 ab
T4	25.5 ± 1.1 ab	7 ± 1.2 b	151 ± 16.8 bcd	1.75 ± 0.57 b
T5	27.1 ± 0.8 ab	8 ± 0.5 ab	161 ± 19.6 bc	1.87 ±0.38 ab
T6	21.6 ± 1.0 b	6 ± 0.8 b	51 ± 7.9 d	1.26 ± 0.41 b
CV (%)	16.7	25.1	26.8	25.1

RF = (number of eggs per root/1000 J2) (100). *n* = 10. Values with the same letter are not significantly different, Tukey, (*p* < 0.05). CV = Coefficient Variation.

**Table 2:** Genome features of *Serratia ureilytica* UTS.

Features	Chromosome
Contigs	134
Genome size	5,401,403 bp
GC content (%)	59.32561
Coding gene	5303
tRNA	87
rRNA	6
Hypothetical proteins	954
Proteins with functional assignments	4349

**Table 3:** Location of genes and gene products involved in nematicidal activity present in the genome of Serratia ureilytica UTS.

Genomic Location	*Serratia ureilytica* UTS Protein
1–6213 87709–93531 3–2393 1–408 3–206	Putative peptide synthetase, containing non-ribosomal peptide synthetase
117538–118815	Chitinase (A)
76512–78203	Chitinase (A)
7930–9444	Serine 3-dehydrogenase
56070–57587	Colicin V production protein
64025–64888	Pyoverdine biosynthesis protein
38269–39687	Serine 3-dehydrogenase
36474–38090	Protease C
50375–51874	Chitinase (B)
49385–50767	Matrixin/peptidase M10 serralysin
58625–59740	Chitinase (C)
23187–28013	Hemolysin
28059–29738	Hemolysin activation protein
7930–9444	Serralysin

## Data Availability

The data from this Whole Genome Shotgun project was deposited in GenBank under the accession number Bioproject PRJNA759051.
